# Phosphorus and sodium contents in commercial wet foods for dogs and cats

**DOI:** 10.1002/vms3.183

**Published:** 2019-07-05

**Authors:** Marcio A. Brunetto, Rafael V. A. Zafalon, Fabio A. Teixeira, Thiago H. A. Vendramini, Mariana F. Rentas, Vivian Pedrinelli, Larissa W. Risolia, Henrique T. Macedo

**Affiliations:** ^1^ School of Veterinary Medicine and Animal Science University of São Paulo São Paulo/Pirassununga Brazil

**Keywords:** minerals, nutritional profile, pet food, phosphate, safe upper limit

## Abstract

It has been reported that wet foods for dogs and cats have high levels of sodium and phosphorus due to their composition. Therefore, this study aimed to evaluate the sodium and phosphorus contents in wet pet foods, and compare it to daily requirements for both species. Twenty‐five commercial wet foods for adult animals were evaluated, 13 for dogs and 12 for cats. The analyses’ results were compared to the European Pet Food Industry Federation (FEDIAF 2018) recommendations. All foods contained phosphorus and sodium amounts above minimum requirements. Three wet foods for dogs exceeded the safe upper limit for phosphorus, and four wet foods for dogs and three for cats exceeded 3.75 g of sodium/1,000 kcal metabolizable energy (ME), considered safe by FEDIAF. No studies were found at the present time evaluating whether higher sodium levels are safe for dogs and cats; however, consumption of high phosphorus diets has been associated with adverse effects on renal function parameters. Therefore, more studies are necessary to investigate the health implications of phosphorus and sodium concentrations observed in some foods evaluated in this research.

## Abstract 2

It has been reported that wet foods for dogs and cats have high levels of sodium and phosphorus due to their composition. Therefore, this study aimed to evaluate the sodium and phosphorus contents in wet pet foods, and compared to daily requirements for both species.

## Graphical abstract

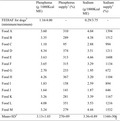

## 1 INTRODUCTION

Studies point out that offering wet foods may be an important way to increase water intake, especially for cats and some small dog breeds, such as Miniature Schnauzer, and this may be a key factor in the prevention and treatment of uroliths (Buckley, Hawthorne, Colyer, & Stevenson, 2011; Seefeldt & Chapman, 1979).

Another way to stimulate water intake is increasing dietary sodium intake (Chandler, 2008; Hawthorne & Markwell, 2004). However, increasing sodium may be contradictory due to its possible harmful effects such as hypertension, progression of renal and heart diseases, hypercalciuria, and increased risk of urolith formation (Nguyen, Reynolds, Zentek, Paßlack, & Leray, 2017). In this context, phosphorus may also be a key factor as its increased intake may be related to increased risk of urolith formation (Lulich et al., 2016) and development of chronic kidney disease (CKD) (Böswald, Kienzle, & Dobenecker, 2018; Dobenecker, Webel, Reese, & Kienzle, 2018), as well as speeding progression of this disease in dogs and cats (Osborne, 1995).

It has been reported that wet foods for dogs and cats have high levels of sodium and phosphorus due to their composition of mainly fresh meats (Crane et al., 2011). However, in the literature, there is only one study that evaluated levels of phosphorus and sodium in wet foods commercialized in the United Kingdom (Davies et al., 2017). These authors observed that concentrations of both minerals were higher in wet foods when compared to dry foods.

Due to a lack of information in the literature regarding the nutritional profile of wet foods, the present study aimed to analyse phos European Pet Food Industry phorus and sodium contents and compare them to FEDIAF (2018) nutritional requirements.

## 2 MATERIAL AND METHODS

### 2.1 Wet foods

Twenty‐five wet foods were selected based on the availability in major pet stores in the cities of Pirassununga and Campinas (Sao Paulo/Brazil). Thirteen of these wet foods were products for adult dogs and 12 were for adult cats, and were all manufactured by 13 different companies. Of the 25 wet foods, 11 were international brands and 14 were national brands.

### 2.2 Chemical analysis

Analyses of sodium and phosphorus contents were performed at the Multiuser Laboratory of Animal Nutrition and Bromatology at the Department of Animal Nutrition and Production of the School of Veterinary Medicine and Animal Science, and the Agrarian Laboratory of the Department of the School of Animal Science and Food Engineering, both of the University of Sao Paulo.

Phosphorus concentrations were measured by spectrophotometry (Caputi, 2013) and sodium concentrations were obtained by atomic absorption spectrophotometry (AOAC, 2007). All analyses were performed in duplicate and repeated when variation over 5% was observed.

Phosphorus and sodium contents were calculated in grams (g) per 1,000 kilocalories (kcal) of metabolizable energy (ME). ME was estimated by the most current method, described in NRC (2006), which consists of different methods for estimating metabolizable energy for dog and cat foods.

## 3 RESULTS

Based on FEDIAF (2018) daily requirements, all 13 wet foods for dogs and 12 for cats presented higher phosphorus concentrations than these recommendations (Tables 1 and 2), considering energy intake of 95 kcal/kg BW^0.75^ for dogs and 100 kcal/kg BW^0.67^ for cats, which are the most commonly used equations to calculate daily energy requirement of these species. Of all foods analysed, 10 for dogs and seven for cats had phosphorus concentrations over twice the recommendations (Tables 1 and 2). Three of the diets for adult dogs presented phosphorus concentrations higher than 4 g/1,000 kcal ME, which is the nutritional limit for healthy adult dogs according to FEDIAF (2018).

**Table 1:** Daily requirements and percentage of supply of phosphorus and sodium for dogs according to FEDIAF (2018)

	Phosphorus (g /1,000 Kcal ME)	Phosphorus supply^a^ (%)	Sodium (g/1,000 Kcal ME)	Sodium supply^a^ (%)
FEDIAF for dogs^b^ (minimum/maximum)	1.16/4.00	–	0.29/3.75	–
Food A	3.60	310	4.04	1,394
Food B	3.35	289	4.38	1,512
Food C	1.10	95	2.88	994
Food D	4.34	374	3.51	1,211
Food E	3.63	313	4.66	1,608
Food F	3.65	315	3.29	1,134
Food G	2.70	233	1.95	672
Food H	4.26	367	3.20	1,104
Food I	1.83	158	2.59	894
Food J	1.64	141	1.87	646
Food K	3.26	281	3.39	1,167
Food L	4.08	351	3.53	1,216
Food M	3.24	279	4.44	1,532
Mean ± *SD* c	3.13 ± 1.03	270 ± 89	3.36 ± 0.89	1,160 ± 306

a Values in percentage refer to how much the food supplies the daily recommendation of the respective nutrient according to FEDIAF (2018).

b Minimum requirements for dogs according to FEDIAF (2018) considering daily energy intake of 95 kcal × kg body weight^0.75^.

c Standard deviation.

**Table 2:** Daily requirements and percentage of supply of phosphorus and sodium for cats according to FEDIAF (2018)

	Phosphorus (g /1,000 Kcal ME)	Phosphorus supply^a^ (%)	Sodium (g/1,000 Kcal ME)	Sodium supply^a^ (%)
FEDIAF for cats^b^ (minimum/maximum)	1.25/−		0.19/3.75	
Food A	2.13	170	1.68	885
Food B	3.55	284	2.39	1,259
Food C	2.74	219	3.11	1,638
Food D	5.29	423	3.07	1,617
Food E	2.25	180	0.86	451
Food F	4.36	349	4.11	2,162
Food G	3.96	317	4.67	2,460
Food H	1.67	134	2.12	1,114
Food I	1.74	139	1.06	557
Food J	4.89	392	5.64	2,966
Food K	1.88	150	1.40	738
Food L	2.64	211	2.97	1,561
Media ± *SD* c	3.09 ± 1.28	247 ± 102	2.76 ± 1.48	1,451 ± 779

a Values in percentage refers to how much the food supplies the daily recommendation of the respective nutrient according to FEDIAF (2018).

b Minimum requirements for cats according to FEDIAF (2018) considering daily energy intake of 100 kcal × kg body weight^0.67^.

c Standard deviation.

Sodium concentrations of all wet foods were above recommended intake according to FEDIAF (2018) (Tables 1 and 2). Nine wet foods for dogs and eight for cats exceeded by more than 10 times the recommendations of sodium (Tables 1 and 2). Seven of the 25 wet foods (four for dogs and three for cats) had sodium amounts higher than 3.75 g/1,000 kcal, the safe upper limit for both dogs and cats established by FEDIAF (2018).

## 4 DISCUSSION

This study showed that high sodium values were present in the wet foods evaluated, and seven of the 25 products had sodium levels above the safe upper limit according to FEDIAF (2018). However, FEDIAF (2018) states that perhaps higher levels may be safe, but no scientific data are available.

Despite the potential benefit of increased water intake associated with higher sodium contents in pet food (Chandler, 2008), excessive ingestion of sodium by humans has been associated with increased risk of developing systemic arterial hypertension, cardiac disease (Institute of Medicine, 2004; Strazzullo, D'Elia, Kandala, & Cappuccio, 2009; WHO, 2007), nephropathies (Ritz, Koleganova, & Piecha, 2009), damage to gastric mucosa and gastric tumours (Kono, Ikeda, & Ogata, 1983; Tsugane & Sasazuki, 2007).

Short‐ and medium‐term studies using either healthy adult cats, healthy older cats and dogs or cats with induced chronic kidney disease did not observe harmful effects of high sodium intake (2.9–3.2 g Na/1,000 kcal) on blood pressure (Buranakarl, Mathur, & Brown, 2004; Cowgill, Segev, & Bandt, 2007; Greco, Lees, Dzendzel, Komkov, & Carter, 1994; Kirk, Jewell, & Lowry, 2006; Xu, Laflamme, & Long, 2009). The absence of negative effects despite high sodium intake may be explained by the presence of mechanisms that control the organism's sodium balance, like the interaction between vascular pressure receptors, renal sympathetic enervation and vasopressin hormone, renin‐angiotensin‐aldosterone system, and natriuretic peptides (Bartges, 2012). This may also explain the range between minimum recommendation (0.29 g/1,000 kcal for dogs and 0.19 g/1,000 kcal for cats) and safe upper limit (3.75 g/1,000 kcal for both dogs and cats) according to FEDIAF (2018).

The effect of high sodium content in pet foods is controversial, especially for pets that have systemic arterial hypertension, and the consensus of management of this disease recommends avoiding the excessive consumption without necessarily indicating decreasing this mineral's intake (Brown et al., 2007).

In older cats, a sodium intake of 3.1 g/1,000 kcal did not increase the risk of myocardial dysfunction or glomerular filtration rate (Chetboul et al., 2014; Reynolds et al., 2013). However, dogs diagnosed with congestive heart failure seem to benefit from diets with low sodium levels (0.4 g/1,000 kcal) when compared to moderate sodium levels (0.7 g/1,000 kcal) (Rush et al., 2000). None of the studies above‐mentioned have used more than 3.75 g of sodium per 1,000 kcal content considered safe by FEDIAF differently from our study, in which seven wet foods exceeded this amount.

The safe upper limit for sodium intake is also suggested by the consensus for treatment and prevention of urolith formation in dogs and cats (Lulich et al., 2016). High sodium intake has been associated with hypercalciuria, which may pre‐dispose patients to formation of calcium and struvite uroliths (Kirk et al., 2006). Lekcharoensuk et al. (2001) observed that out of 290 cats, those who had high sodium intake (1.42–3.71 g/1,000 kcal) for 6 months presented 4.1 times more chances of developing struvite uroliths when compared to cats that had an intake of 0.48–0.77 g/1,000 kcal. However, there was a correlation found between high sodium and high phosphorus contents, which may have influenced the results.

Regarding phosphorus content, in the present study, three wet foods for dogs presented levels above the safe upper limit according to FEDIAF (2018). There is no established safe upper limit by FEDIAF (2018) for phosphorus in cats.

As water intake by consuming wet foods is increased which therefore decreases dehydration and increases diuresis, this type of food may be prescribed for patients with uroliths (Buckley et al., 2011; Seefeldt & Chapman, 1979). However, of the 25 wet foods evaluated in this study, three products for dogs had phosphorus levels above the safe upper limit recommended by FEDIAF (2018), which can be pre‐judicial because high phosphorus intake is associated with struvite uroliths and may even make it harder to dissolve these uroliths (Abdullahi, Osborne, Leininger, Fletcher, & Griffith, 1984; Osborne, Lulich, Forrester, & Albasan, 2009).

The consumption of high phosphorus diets is associated with adversely affecting renal function parameters in healthy cats (Dobenecker et al., 2018) and also with an increased risk of developing chronic kidney disease (CKD) in this species (Böswald et al., 2018). Dobenecker et al. (2018) observed that cats consuming a diet with a phosphorus content five times higher (1.6% phosphorus in dry matter) than the minimum requirement had decreased creatinine clearance and increased renal phosphorus excretion when compared to the control group which consumed a diet with 0.56% of phosphorus in dry matter. These authors also observed glycosuria and microalbuminuria in nine of the 13 cats in the high phosphorus diet group. In a retrospective study, Böswald et al. (2018) observed that cats with CKD consumed a greater amount of phosphorus in the period prior to diagnosis, compared to older cats who did not present with CKD.

It is important to consider the type of phosphorus involved when establishing and evaluating its content in foods, because organic and inorganic forms have different physiological effects. While the organic form binds to proteins resulting in lower absorption, the inorganic phosphorus is absorbed much more readily, making its excess potentially more harmful to animals. Coltherd et al. (2018) observed that addition of inorganic phosphorous salts in the diet at levels from 0.5 g/1,000 kcal is able to increase plasma phosphorus in cats. Meanwhile, the authors did not verify changes in plasma phosphorus concentration after the ingestion of organic phosphorus. A harmful effect of the inorganic form of phosphorus was described by Alexander et al. (2018) in a study evaluating high inclusion of inorganic phosphorus in a diet for healthy cats. Authors observed loss of renal function and changes in echogenicity suggestive of renal pathology, observed by ultrasonography, after high phosphorus intake.

In dogs, Herbst and Dobenecker (2018) also observed that diets containing inorganic sources of phosphorus (2.1% in dry matter) were more efficient in increasing postprandial serum concentrations of phosphorus and parathyroid hormone (PTH) when compared to organic sources, as well as in increasing the area under the curve of PTH. Dogs fed diets with inorganic phosphorus sources had calcium vs. phosphorus serum product exceeding 2.1 times the recommended (55 mg^2^/dl^2^).

Furthermore, hyperphosphataemia is related to progression of CKD, because it stimulates parathyroid hormone secretion and consequently secondary renal hyperparathyroidism (SRHP), which increases nephron loss (Osborne, 1995). Several studies showed that phosphorus restriction is beneficial to dogs and cats with induced and naturally acquired CKD (Barber, Rawlings, Markweu, & Elliott, 1999; Bonder et al., 2016; Brunetto et al., 2016; Lopez‐Hilker, Dusso, Rapp, Martin, & Slatopolsky, 1990; Ross et al., 2006; Ross, Finco, & Crowell, 1982; Slatopolsky et al., 1971). Moreover, there is evidence that dogs and cats with low phosphorus intake had an increase in survival time (Elliott, Rawlings, Markwell, & Barber, 2000; Jacob et al., 2002; Ross et al., 2006).

The limitations of the present study are the small number of foods evaluated, the fact that calcium content of the products was not analysed, and estimating metabolizable energy with the use of a prediction equation rather than analysing.

## 5 CONCLUSION

High phosphorus and sodium concentrations were observed in most of the evaluated foods. Some of them exceeded safe upper limits established by the European Pet Food Industry (FEDIAF 2018). With regard to phosphorus, this high content might be harmful when consumed in long term. On the other hand, in relation to sodium, further studies are needed to investigate whether concentrations found in this study are safe for dogs and cats. Therefore, several wet foods evaluated in the present study are not recommended, although available in the pet food market. This study provides a stimulus for the pet food industry to review its formulations and inclusion of mineral supplements as well as to promote constant assessments of their products.

## CONFLICT OF INTEREST

The authors declare that they have no conflict of interest.

## ETHICAL APPROVAL

The authors confirm that the ethical policies of the journal, as noted on the journal's author guidelines page, have been adhered to. No ethical approval was required as this is an article that does not use animals or their tutors.

## CONTRIBUTIONS

Study design was performed by R.V.A.Z. and M.A.B. All authors participated in the manuscript writing and review process. Laboratorial analyses were performed by R.V.A.Z.
